# The Effect of Deposition Time on the Surface Coverage of Sublimation Deposited Solid-Phase Glycine and Proline Molecules Measured by Scanning Tunneling Microscopy

**DOI:** 10.3390/molecules25132962

**Published:** 2020-06-28

**Authors:** Young-Sang Youn

**Affiliations:** Department of Chemistry, Yeungnam University, Daehak-ro 280, Gyeongsan, Gyeongbuk 38541, Korea; ysyoun@yu.ac.kr; Tel.: +82-53-810-2357

**Keywords:** coverage, deposition time, solid-phase molecules, scanning tunneling microscopy, Ge(100)

## Abstract

The effect of deposition time on the surface coverage of sublimation deposited solid-phase glycine and proline molecules onto a Ge(100) surface was studied at room temperature using scanning tunneling microscopy (STM). The STM images obtained at various coverages of glycine and proline adsorbed on the Ge(100) surface showed that (i) the adsorption rate for both molecules gradually decreased with increasing deposition time, obeying the Langmuir adsorption model, and (ii) the coverage of glycine on the Ge(100) surface is higher than that of proline under the same deposition conditions, which may be due to the differences in their molecular weight or molecular sticking probability.

## 1. Introduction

Research on the interactions formed between organic/biomolecules and semiconductor surfaces has been extensively carried out toward the development of their industrial application in various fields [1,2,3,4,5,6,7]. In particular, understanding the adsorption structure and reaction mechanism of molecules deposited on group IV (100) semiconductor surfaces, which are most commonly used as basic starting materials in semiconductor-based industries, is important in terms of extending the fundamental knowledge on semiconductor surface reactions [8,9,10].

When a Ge crystal with a diamond structure is cut along the plane of the (100) direction, all of the surface Ge atoms would have two dangling bonds. Since these bonds are unstable, surface reconstruction occurs to minimize the surface energy, resulting in the dimerization of two dangling bonds [11,12]. Consequently, surface periodicity along the dimer direction is doubled, and domains of symmetric (2 × 1) and asymmetric (4 × 2) reconstructions are formed.

Studies on gas- and liquid-phase molecules on Si(100) or Ge(100) surfaces have been extensively reported in the literature [13,14,15]. In addition, studies on solid-phase molecules have also been conducted, although they are difficult to carry out experimentally [16,17,18,19,20]. The Langmuir (L) is the useful unit used to measure the amount of gas- or liquid-phase molecules deposited onto a surface when the experiment is performed under ultra-high vacuum (UHV) and is calculated as the product of the deposition pressure and exposure time (1 L = 10^−6^ Torr∙s). The amount of gas- or liquid-phase molecules deposited onto a surface can be quantified using this unit of measurement, while that of solid-phase molecules is difficult to quantify because they must be sublimed at an appropriate temperature, which is set depending on the experimental conditions used.

**Figure 1:**
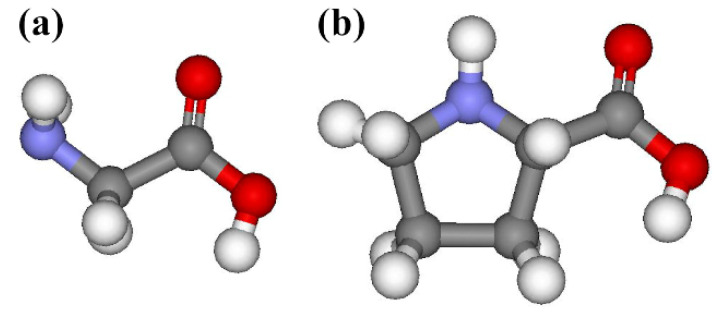
Schematic representations of (**a**) glycine and (**b**) L-proline. The red, blue, grey, and white balls indicate oxygen, nitrogen, carbon, and hydrogen atoms, respectively.

Scanning tunneling microscopy (STM) can be used to investigate the relationship between the coverage and deposition time of solid-phase molecules because it can visualize the molecules adsorbed on a surface and used to count the number of the adsorbed species [11,21,22]. Herein, solid-phase glycine and proline molecules (Figure 1) with simple molecular structures were explored because their adsorption structures on a Ge(100) surface were known as the features exhibiting a bright protrusion in filled-state STM images [17,18]. Hence, abundant STM images obtained at various coverages of glycine and proline adsorbed onto a Ge(100) surface were analyzed by counting the number of bright protrusions, which represent an individual adsorbed molecule, to demonstrate the relationship between the coverage and deposition time. It was discovered that the adsorption rate gradually decreases in both glycine and proline upon increasing the deposition time. Furthermore, the coverage of glycine adsorbed onto the Ge(100) surface is higher than that of proline under the same deposition conditions. Based on these results, it is expected that the adsorption coverage of solid-phase molecules on the Ge(100) surface can vary depending on the type of molecules used even under the same deposition conditions.

## 2. Materials and Methods

The Ge(100) surface was cleaned via repeated sputtering cycles using 1 keV Ar^+^ ions at 700 K, followed by annealing at 900 K in an UHV chamber. The cleanliness of the Ge(100) surface was confirmed using STM. Glycine (NH_2_CH_2_COOH, 98.5% purity, Sigma-Aldrich, Seoul, Korea) and L-proline (C_4_H_7_NHCOOH, 98.5% purity, Sigma-Aldrich, Seoul, Korea) were individually purified via several sublimation and pumping cycles to remove all dissolved gases prior to deposition. The deposition of the solid-phase glycine and proline molecules onto the Ge(100) surface at room temperature was conducted using a home-built solid-phase molecule deposition instrument, which consists of three components: a glass tube wrapped with a heating wire, kovar glass for loading the solid-phase sample, and a gate valve for connecting the glass tube and kovar glass. For the sublimation of the molecules, the glass tube and kovar glass components of the home-built solid-phase molecule deposition instrument were heated at 420 and 320 K during the deposition process, respectively.

STM experiments were carried out in an UHV chamber equipped with an OMICRON STM instrument (Berlin, Germany) at a base pressure below 1.2 × 10^−10^ Torr. The filled-state STM images were obtained using an electrochemically etched tungsten tip at a bias voltage (V_s_) of −2.0 V and tunneling current (I_t_) of 0.1 nA. Herein, the coverage was expressed using the monolayer (ML) unit, which was determined by dividing the total number of adsorbed molecules by the number of dimers consisting of two Ge atoms. Because the major adsorption structure for both the glycine and proline molecules on the Ge(100) surface, which is an “intrarow O-H dissociated and N dative bonded structure”, was produced upon the reaction with two Ge dimers, the maximum value of coverage will be 0.500 ML without considering their minor adsorption features. All coverage values calculated from the STM images have an error rate, which gives a 95% confidence interval.

## 3. Results

**Figure 2:**
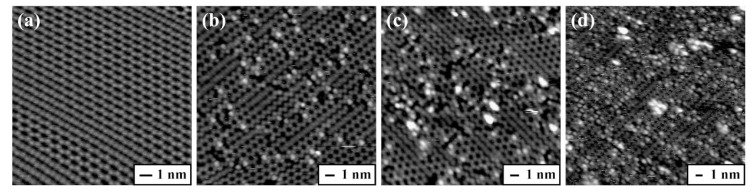
Representative filled-state STM images of (**a**) clean Ge(100) surface, (**b**) 0.054, (**c**) 0.083, and (**d**) 0.141 ML glycine adsorbed onto the Ge(100) surface at room temperature. The individual deposition times obtained under the same deposition conditions are (**b**) 120, (**c**) 180, and (**d**) 600 s. The adsorbed glycine on the Ge(100) surface appears as a bright protrusion in the STM images. The scan area of the STM images are (**a**) 14.1 × 14.1, (**b**) 18.6 × 18.6, (**c**) 20.0 × 20.0, and (**d**) 25.6 × 25.6 nm^2^.

Figure 2 displays the filled-state STM images obtained before and after the deposition of glycine onto a Ge(100) surface over 120, 180, and 600 s at room temperature. The adsorption feature of glycine on the Ge(100) surface appears as a bright protrusion between two dimer rows with a dark adjacent dimer in the STM images, which was named as an “intrarow O-H dissociated and N dative bonded structure” [17]. Therefore, the number of the bright protrusions observed in the STM images was used to calculate the adsorption coverage of glycine on the Ge(100) surface. When the exposure time for glycine deposition onto the Ge(100) surface was 120, 180, and 600 s, the coverage was determined to be 0.054 ± 0.003, 0.083 ± 0.007, and 0.141 ± 0.008 ML, respectively upon analyzing the STM images.

**Figure 3:**
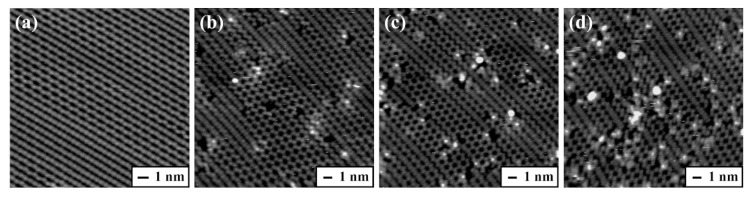
Representative filled-state STM images of (**a**) clean Ge(100) surface, (**b**) 0.016, (**c**) 0.035, and (**d**) 0.066 ML proline adsorbed onto the Ge(100) surface at room temperature. The individual deposition times under the same deposition conditions are (**b**) 60, (**c**) 180, and (**d**) 480 s. The adsorbed proline on the Ge(100) surface appears as bright protrusions in the STM images. The scan area of the STM images are (**a**) 16.0 × 16.0, (**b**) 20.0 × 20.0, (**c**) 20.0 × 20.0, and (**d**) 20.0 × 20.0 nm^2^.

Figure 3 describes the filled-state STM images taken before and after the deposition of proline onto the Ge(100) surface over 60, 180, and 480 s at room temperature. The adsorption coverage of proline was calculated in the similar way to glycine because all three of the distinct adsorption configurations of proline on the Ge(100) surface exhibit bright protrusions, which were designated as an “intrarow O-H dissociated and N dative bonded structure”, “O-H dissociation structure”, and “N dative bonded structure”, respectively [18]. On the basis of the analysis of the STM images obtained at a deposition time of 60, 180, and 480 s, the coverage of proline adsorbed onto the Ge(100) surface was determined to be 0.016 ± 0.003, 0.035 ± 0.007, and 0.066 ± 0.005 ML, respectively.

**Figure 4:**
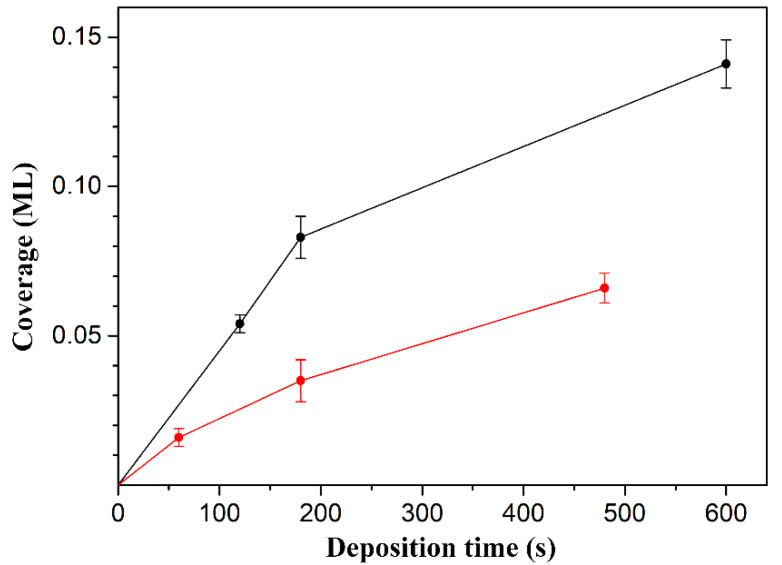
The average coverage as a function of the deposition time of glycine (black) and proline (red) molecules onto the Ge(100) surface. The error bars exhibit the 95% confidence interval.

To clearly identify the relationship between the coverage and deposition time, the coverage values and their corresponding error bars obtained from the statistical analysis of the STM images were plotted, as shown in Figure 4. Figure 4 shows two interesting features. First, the adsorption rate of the molecules on the Ge(100) surface gradually decreases upon increasing the deposition time. Using the shapes of the molecular adsorption rate curves, they appear to obey the Langmuir adsorption model within these coverages, in which the adsorption rate depends on the number of vacant sites on the surface because the number of empty sites available for adsorption decreases as the coverage increases [23,24]. Second, the coverage of glycine was higher than that of proline even under the same deposition conditions, which may be caused by the difference in their molecular weight or molecular sticking probability. In particular, it is expected that the amount of sublimed glycine was high when compared to that of proline under the same deposition conditions because the molecular weight of glycine (Mw = 75) is smaller than that of proline (Mw = 115) [25]. From the point of view of the sticking probability, it is expected to be similar because the molecular structures of both molecules are similar, except that the functional group in glycine is a primary amine and proline is a secondary amine.

## 4. Conclusions

The relationship between the surface coverage and deposition time during the sublimation deposition of solid-phase glycine and proline molecules onto a Ge(100) surface at room temperature was investigated using STM. Analysis of the STM images obtained at various coverages depending on the deposition time onto the Ge(100) surface showed that the adsorption rate of the molecules gradually decreased upon increasing the deposition time, obeying the Langmuir adsorption model within these coverages. Moreover, under the same deposition conditions, the adsorption rate of glycine is faster than that of proline on the Ge(100) surface, indicating that the coverage of the solid-phase molecules onto the Ge(100) surface depends on the type of molecule used.
